# Supplementary material for: Are carbon emissions trading and green financial instruments synergistic? -Comprehensive quantitative research based on content analysis
Source: PLoS One. 2024 Mar 7;19(3):e0298601. doi: 10.1371/journal.pone.0298601 (PMC10919601; doi:10.1371/journal.pone.0298601)
Supplement: S1 Data — (ZIP) [file pone.0298601.s001.zip › Data/地级市Z_F工作报告.zip!/┴╔─■/║∙┬1⁄2╡║╩╨/└┤╫╘╣1⁄2╓┌║┼í╛┬φ┐╦╩2╛▌═°í┐_2014.docx]

各位代表：

　　现在，我代表市人民政府向大会报告工作，请予审议，并请各位政协委员和其他列席同志提出意见和建议。

　　一、过去四年工作回顾

　　过去的四年，是经济社会形势复杂、矛盾凸显的四年，也是全市发展励精图治、稳中有为的四年。四年来，面对宏观经济下行的巨大压力和改革发展稳定的诸多困难，在市委的坚强领导下，全市人民团结一心，攻坚克难，圆满完成了市四届人大一次会议确定的各项目标任务。

　　预计2013年，地区生产总值784亿元，增长9%，是2009年的1.8倍；公共财政预算收入88.4亿元，增长5.2%，是2009年的2倍；固定资产投资680亿元，增长25%，是2009年的2.7倍；社会消费品零售总额370亿元，增长13.5%，是2009年的1.9倍；城镇居民人均可支配收入25200元，增长10%，是2009年的1.6倍；农民人均纯收入10070元，增长12%，是2009年的1.8倍。经济社会保持了平稳较快发展，主要体现在8个方面：

　　1、工业经济持续向好，项目建设成效显著。2013年，规模以上工业总产值1050亿元，是2009年的1.5倍；规模以上工业增加值220亿元，是2009年的1.5倍。规模以上工业企业392家，销售收入超10亿元企业8家，是2009年的1.2倍。锦西石化销售收入超300亿元，渤船重工销售收入超100亿元。

　　结构调整实现较快进展。2013年，数字技术、泵阀等6大产业集群企业超过1000家，是2009年的2倍。5个产业集群列入省重点，4个产业集群销售收入突破100亿元，泳装产业跻身中国服装产业十大示范集群行列。高新技术产品增加值34亿元，是2009年的2.2倍。实施技术改造项目145个，是2009年的2倍。四年来，并购海外企业13家，引进国际知名品牌17个，获得省级以上科技成果43项。锦化研究院“大型客机窗用玻璃板材”填补了国内空白，渤船重工“AP1000核电管道”被评为国家战略性创新产品，西门子工业透平SST－400型汽轮机实现首台国产化。

　　项目年活动成效明显。2013年，实施亿元以上项目255个，总投资2350亿元，分别是2009年的2.6倍和1.4倍。四年来，共实施亿元以上项目420个，总投资3600亿元。投资78亿元的绥电二期、投资6亿元的10万吨糠醇等187个重点项目建成投产。投资100亿元的葫芦岛铝业完成厂房基础建设，徐大堡核电、大唐热电、渤船重工海工等重大项目加快推进。

　　2、县域经济提速发展，民营经济活力增强。2013年，县域生产总值375亿元，是2009年的2.2倍；公共财政预算收入36.7亿元，是2009年的3.1倍。粮食产量连创4个丰收年，绥中连续6年被列为国家产粮油大县，兴城连续3年被列为国家产粮油大县，建昌被列为2013年国家产粮大县。水果、蔬菜、肉蛋奶产量分别是2009年的1.5倍、2倍和2.6倍。县城面积扩大到71平方公里，城镇化率51.2%，比2009年提高15个百分点。

　　现代农业长足发展。11个投资10亿元以上农产品深加工项目开工。实施节水滴灌面积62万亩，累计建设设施蔬菜140万亩，是2009年的2.8倍；建设畜禽标准化养殖小区718个、工厂化渔业180万平方米，分别是2009年的2.3倍和1.8倍，绥中白梨列入省一县一业。农民专业合作社发展到1817家，是2009年的2.8倍。治理水土流失150万亩，完成农建骨干工程1550项，连续2年获省农建大禹杯，建昌县荣获全国农田基本建设先进县。建设农村饮水安全工程1230处，提前2年完成“十二五”任务。

　　民营经济活力逐步释放。召开了全市中小微企业发展大会，成立了市中小企业服务中心，制定了系列扶持政策，设立了专项资金，搭建了融资保障平台，推动了民营经济的发展。2013年，全市民营经济增加值345亿元，占全市地区生产总值的44%，是2009年的1.6倍；营业收入超亿元的民营企业167家，是2009年的1.7倍。45家企业获得全省民营明星企业称号。

　　3、城区经济繁荣兴旺，服务业全面发展。2013年，城区生产总值381亿元，是2009年的2.4倍；公共财政预算收入40.9亿元，是2009年的1.5倍。完成连山和南票区划调整，下放经济管理权限65项，增强了城区经济活力。2013年，全市服务业增加值321亿元，增长10.3%，是2009年的1.7倍。

　　“一主四辅”服务业集聚区加速形成。合富广场等50个亿元以上项目开工，大润发、沃尔玛、特易购、中旺百货开业。全市大型商业网点达到104个，玉皇商城、葫芦山庄跻身全省100家服务业领军企业，连山五金机电城、龙港文萃路购物街、龙港兴隆大家庭列入全省100个服务业重点项目。龙港、连山、建昌商贸集聚区和万家数字技术产业基地、觉华岛旅游度假区成为省级服务业集聚区。

　　现代服务业加快发展。银行机构达到20家，是2009年的2倍；各类金融机构达到82家，是2009年的2.5倍；成功发行2期共38亿元城投债券，实现融资渠道多元化。2013年，存款余额1200亿元，是2009年的1.7倍；贷款余额733亿元，是2009年的1.6倍。新增贷款95.3亿元，创历史新高。房地产业开发投资176亿元，增长30%，是2009年的4.3倍；商品房销售额135亿元，增长60%，是2009年的1.7倍，增长速度连续3年位居全省前列。

　　4、沿海经济带活力倍增，对外开放成果显著。2013年，园区七通一平面积115平方公里，是2009年的1.9倍；建成和在建项目313个，完成投资643亿元，是2009年的3.8倍；园区生产总值128亿元，是2009年的3.8倍；公共财政预算收入17.9亿元，是2009年的5倍。园中园起步建设，引进工业地产项目21个，总投资407亿元。环球之星产业园一期基本建成，侨商新材料、青牛软件等7个产业园加快建设，东戴河·中关村科技成果产业化基地完成规划，成为首个中关村直接投资的全国区域合作项目。打渔山等5个园区被认定为国家和省级特色产业基地，东戴河新区建设成为全省样板。

　　开放水平大幅提升。2013年，实际利用外资6.45亿美元，增长21.7%，是2009年的9.7倍；引进内资315亿元，增长26%，是2009年的3.4倍；外贸出口10.8亿美元，增长13.5%，是2009年的2.2倍。成功举办三届泳装文化博览会和泵阀产品博览会，中国葫芦岛·兴城国际沙滩·泳装文化博览会跻身2013中国十大最具发展潜力展会，船舶和泳装被评为省级出口基地。

　　5、文化旅游业纵深推进，新型支柱产业迅速兴起。科学开发海滨、温泉、乡村、古城、长城和岛屿文化旅游资源，形成了“五线一岛”文化旅游品牌。2013年接待游客2526万人次，增长25%，是2009年2.3倍；旅游收入271亿元，增长25%，是2009年的3.6倍。

　　旅游设施加快建设。总长17公里的东戴河、觉华岛、兴海、龙湾4条海滨观光木栈道建成使用，塔山阻击战纪念馆开馆。六股河漂流、灵山森林公园等17个乡村旅游项目投入运营，世界温泉部落、首山·丽汤等3个温泉旅游项目主体完工，小虹螺山、画廊谷等6个沟域旅游项目启动。4A级景区达到6个，旅行社达到83家，旅游宾馆酒店达到455家，新开业五星级酒店2家。

　　文化遗产保护利用稳步推进。沙锅屯遗址、东大杖子战国古墓葬群等7处文化遗址成为全国重点文物保护单位，总数达到13处。姜女石遗址、兴城古城、圣水寺和明代长城列为国家“十二五”重大文化遗产保护项目，兴城古城展示利用加快推进，建昌古生物化石在北京展出。兴城满族秧歌、建昌灯会等5个项目成为省级非物质文化遗产。

　　文化创意产业高点起步。成功举办海上传奇·北方创意经济峰会和创意驱动城市发展论坛，葫芦烙画成为全国葫芦文化地理标志商标，千名鼓手合奏等3项活动创吉尼斯世界纪录。连山被评为中国民间文化艺术之乡，我市被评为最美中国·文化旅游目的地城市。

　　6、基础设施建设全面推进，生态文明建设成绩斐然。城市基础设施不断完善，葫兴一体化的格局初步形成。青山水库基本完工并蓄水试运行，猴山水库前期工作基本完成并顺利开工，困扰多年的城市缺水问题即将得到根本解决。建兴高速完成工程总量的74%，东戴河新区动车站完工，绥中港建设完成投资20亿元。CBD一场五馆全部竣工，城市道路、广场、管网设施条件大大改善。新建和改扩建公路893条，公路总里程达到6774公里，“四横五纵”交通路网基本形成，连续2年获省文明公路市称号。

　　城乡环境整治逐步深入。实施交通整治三大战役，依法取缔营运三轮车和小中巴，完成公汽公司重组，建立了大公交体系。实施绿化、美化、亮化、净化工程，城中村改造全面启动，消除“摸黑路”35条，荣获全省绿叶杯和城市“四化”竞赛双连胜。实施青山、碧水、蓝天工程，造林绿化375万亩，是2009年的2.3倍。全面启动国家森林城市创建工作，辽西绿色生态长廊工程建设完工。森林覆盖率35.4%，比2009年提高5.8个百分点，荣获省绿化造林先进市三连冠。虹螺山、白狼山晋升国家级自然保护区，龙潭大峡谷成为省级地质公园，白狼山成为省生态文化教育示范基地。建成省级生态乡镇3个，省级生态村33个。凌河生态治理工程竣工，近岸海域水质基本达标。空气优良天数保持在350天以上，兴城被评为全国空气质量最好小城。

　　7、民生质量不断改善，社会事业蓬勃发展。四年来，累计支出民生资金306亿元，占全市财政预算支出的62.2%，市政府向群众承诺的35件实事全部兑现。实名制就业23.2万人，城镇登记失业率控制在3.8%以内，我市就业工作经验被国家人社部总结推广。新农保参保率93.7%；新农合参合率96%，补偿比例最高达80%。3次提高低保标准，15.4万城乡低保对象基本生活得到保障。扎实推进扶贫开发，41.2万农村贫困人口稳定脱贫。启动农村居民大病保险，实施22项重大疾病医疗保障。新建廉租住房1.2万套、经济适用房2480套，8849户农村危房改造全面动工；全国最大的单体棚户区改造项目打渔山南票新城全面完工，6.3万居民即将喜迁新居。社会福利机构增加到27所，90岁以上高龄老人生活补贴标准提高1倍。老城区基本实现24小时供水，消除了供暖集中连片低温区。

　　统筹发展社会事业。全面免除城乡学生义务教育阶段学杂费，新增城乡幼儿园120所。北京语言大学、辽宁省实验中学落户东戴河；锦西工业学校被确定为国家级示范性中等职业学校；渤海船院新校园完工，被评为国家骨干高职院。建成省级文化先进乡镇（街道）、村（社区）58个，文化体育场馆全面投入使用，农家书屋覆盖全市行政村，形成了读书节、文化大讲堂等品牌群众文化。全面实施国家基本药物制度和基层医疗卫生单位综合改革，83家基层医疗卫生单位全部实行网上采购药品，基层卫生服务网络覆盖率100%。盛京医院落户东戴河。人口出生率、自然增长率分别控制在6.63‰和1.66‰以内，荣获省人口和计划生育综合改革示范市。成功承办第十二届全运会足球、蹦床赛事，兴城荣获全国群众体育先进县称号。

　　国防动员、军民共建、民兵预备役等工作成绩显著，荣膺全国双拥模范城四连冠、省双拥模范城五连冠。人防工作荣获全国先进单位称号。国土资源、民族宗教、妇女儿童、新闻出版、外事侨务、国家安全、海关海事、监察、对台、物价、统计、质监、供销等工作不断加强，档案、气象、电力、慈善和红十字会等事业取得新成绩。

　　8、民主法制和精神文明建设得到加强，社会和谐稳定。自觉接受市人大的法律监督、工作监督和市政协的民主监督，坚持定期向市人大报告工作，向市政协通报工作，主动邀请市人大、市政协领导列席市政府常务会议和重要工作会议。认真办理和采纳人大代表、政协委员的意见、建议，积极加强与民主党派、工商联、无党派人士、各人民团体的联系。四年来，办理人大代表建议298件、政协委员提案1892件，办复率、见面沟通率100%，满意和基本满意率99.8%，落实和基本落实率64.6%。积极推行政务公开，41家单位251项行政审批项目网上公开。开展了软环境综合治理活动，六五普法和法治城市创建工作稳步实施，审计监督不断强化，依法行政水平持续提升。社会预警体系和应急管理机制逐步完善，防灾减灾能力明显提升。强化矛盾纠纷调处，积案化解率达86.2%。平安葫芦岛稳步推进，社会治安防控体系逐步完善，蝉联全国社会管理综合治理优秀市两连冠和全省综治工作先进市七连冠，荣获全国平安铁路示范市和见义勇为优秀市三连冠。严厉打击食品药品领域违法犯罪，人民身心健康得到有效保障。安全生产形势全面好转，连续6年荣获全国安全生产月活动优秀单位，连续4年荣获省安全生产工作先进市。

　　深入贯彻党的十八大精神，边学边改开展教育实践活动，扎实开展中国梦教育、社会主义核心价值观教育和文明城市创建活动，讲文明、树新风深入人心，公民道德建设“五个一工程”卓有成效，形成了良好社会风尚，城市文明程度显著提升。

　　各位代表！这些成绩的取得，是省委、省政府和市委正确领导的结果，是市人大、市政协监督支持的结果，是历届班子和老领导、老同志打下良好基础的结果，是全市人民共同努力的结果。在此，我代表市第四届人民政府，向全市广大干部群众，向各位人大代表、政协委员和老领导、老同志，向各民主党派、工商联、人民团体和中省直单位，向驻葫部队、武警官兵和各界朋友，致以崇高的敬意和衷心的感谢！

　　各位代表！过去四年的奋斗历程令人难忘，全市经济社会的发展势头催人奋进。但我们也清醒地看到，我市发展仍然面临不少困难和问题。主要是：经济总量较小，整体经济实力有待提升；新兴产业发展不快，产业结构调整任务繁重；财政收支矛盾突出，经济发展方式转变迫在眉睫；城镇化水平不高，资源和环境压力加大；部分干部责任意识和服务能力不强，干事创业的氛围还不浓厚。对于这些问题，我们一定高度重视，以改革的精神、务实的态度、创新的举措认真加以解决。

　　二、今后四年的目标和任务

　　各位代表！党的十八届三中全会描绘了全面深化改革的宏伟蓝图，为我市未来改革发展指明了方向。我们要站在新起点，瞄准新目标，实现新跨越，开创新局面。

　　今后四年政府工作总体思路是：全面贯彻落实党的十八大和十八届三中全会精神，以科学发展为主题，以结构调整为主线，以深化改革为动力，深入实施工业强市、项目支撑、沿海开放、民生保障四大战略，着力激发市场活力，提高经济发展质量，繁荣各项事业，增进人民福祉，建设生态文明，促进社会和谐，努力建设沿海经济强市。

　　今后四年的预期发展目标是：到2017年末，地区生产总值达到1100亿元，年均增长9%；公共财政预算收入达到125亿元，年均增长9%；固定资产投资达到1500亿元，年均增长20%。

　　按照上述目标要求，重点抓好以下5个方面工作：

　　——全面深化改革，增强加快发展的活力。转变政府职能，深化行政体制改革，简政放权，提高效率。深化财税体制改革，建立事权与支出责任相适应的财政制度。完善现代企业制度，鼓励民资进入公共服务领域。处理好政府和市场、政府和社会中介组织的关系，更好发挥市场在资源配置中的决定性作用，更好发挥政府作用。创新行政管理方式，增强政府公信力和执行力，建设法治政府和服务型政府。

　　——拓展开放领域，加快结构调整步伐。坚持以开放促改革，以园区和港口为龙头，招商引资与域外投资相结合，打造对内对外开放新优势。加快港口建设，实现港城互动、园城互动、产城互动，构建横贯沿海、联结腹地的全方位开放新格局。以城区老工业区搬迁改造为牵引，建设新型产业基地，打造5个产值超100亿元的企业、5个产值超500亿元的产业集群，持续优化产业格局。

　　——统筹区域发展，推进新型城镇化。以新型城镇化为引领，推动城乡要素平等交换、公共资源均衡配置。推进产业集聚，优化城市空间结构，增强城市综合承载能力。发展现代农业，构建新型农业经营体系，推进农业转移人口市民化，实现城镇基本公共服务常住人口全覆盖。县城面积达到100平方公里，主城区面积达到200平方公里，城镇化率达到58%，形成南票、连山、龙港、兴城四位一体的沿海带状大城市格局。将建昌建设成特色产业基地和秀美山城，将东戴河打造成国际化现代生态科技滨城。

　　——建设生态文明，打造美丽葫芦岛。坚持环境优先、生态立市，划定生态保护红线，完善生态治理、生态修复和生态补偿制度，推进绿色发展、循环发展、低碳发展。坚持依法保护环境，单位生产总值能耗持续下降，主要污染物排放总量持续削减。完成国家森林城市创建工作，森林覆盖率达到39%，让生态环境更适宜人居。

　　——改善和保障民生，提前实现全面建成小康社会目标。突出就业创业富民，新增就业15万人，城镇登记失业率控制在4%以内。积极应对老龄化，大力发展养老事业、养老产业。覆盖城乡的社保体系基本建成，教育、文化、医疗等公共服务更趋均等化，非基本公共服务更加丰富，城乡居民人均收入比2010年翻一番，提前全面建成小康社会。

　　三、2014年主要工作

　　今年是贯彻落实党的十八届三中全会精神、全面深化改革的开局之年，也是市委确定的改革创新年。面对错综复杂的经济形势，加快发展仍是解决我市所有问题的关键。我们必须以富民强市为目标，以稳中求进为总基调，稳增长，调结构，促改革，惠民生，不畏艰险，勇于创新，推动经济社会持续健康发展。

　　今年经济发展的主要预期目标是：地区生产总值增长9%，公共财政预算收入增长10%，固定资产投资增长23%，社会消费品零售总额增长13.7%，城镇居民人均可支配收入增长11.5%，农民人均纯收入增长13%。

　　为实现上述目标，重点抓好8个方面的工作：

　　1、推进沿海开发开放。加快招商引资步伐。坚持以工业地产为核心，以北京产业转移、建设“京津冀经济走廊”为契机，实行以商招商、产业链招商。实施工业地产园项目21个，面积20平方公里，力争10个产业园竣工，100家企业落户，东戴河·中关村科技成果产业化基地建设全面启动。全年实际利用外资增长10%，引进内资增长20%。推进出口基地建设，外贸出口增长13.7%。

　　完善园区基础设施。加快推进园区七通一平，年内达到130平方公里。积极创造条件，加快柳条沟、绥中两大港区建设。柳条沟港石化码头试运行，4个10万吨级通用泊位建成使用，疏港铁路开工建设；绥中港2个5万吨级通用泊位建成使用，疏港铁路、疏港公路加快建设；启动临港工业园、物流园建设。加强园区创业大厦、企业孵化和综合服务平台建设，加快职业技术培训基地和生态保护设施建设。强化园区科技支撑，推进校企合作，促进各大企业与大专院校、科研院所联姻，引导科研机构进入园区发展，形成港区一体、港城互动的发展格局。

　　深化园区体制改革。加快园区管理、用人和分配等制度改革，让园区行使更大的经济管理权限。创新综合服务模式，实现商务信息化、贸易便利化。

　　2、加快工业结构调整。完善市领导帮扶重点企业责任制。建立重点企业经济运行分析调度制度，支持重点企业适销对路产品满负荷生产，帮助停产企业逐步恢复生产，提高工业经济运行质量。

　　深入开展项目年活动。积极推进城区老工业区搬迁改造和企业产品结构升级换代，促进产业结构调整。实施亿元以上工业项目155个，总投资2678亿元，积极打造数字技术、聚氨酯、装备制造、泵阀和泳装等工业产业集群。投资410亿元的徐大堡核电站一期、投资33亿元的大唐热电、投资5亿元的高桥风电等42个项目开工建设。投资100亿元的葫芦岛铝业、投资60亿元的渤船重工海洋工程、投资50亿元的中油天宝石油钢管制造、投资1.2亿欧元的梅迪亚斯普游艇制造等113个项目加速建设。投资10亿元的铜铝复合材料、投资5亿元的泰山石膏等55个项目投产达效。投资100亿元的康乃尔80万吨MDI、投资100亿元的中关村科技成果产业化基地、投资40亿元的水利部新华泵阀园等55个项目加快推进。

　　积极推进创新驱动。支持渤船重工发展海洋工程、核电设备产业，推进锦西石化汽柴油质量升级。扶持高新技术企业成长，新增国家级高新技术企业10家，专利申请量增长10%，专利产业化率达到50%，高新技术产品增加值增长25%，规模以上工业增加值增长10%。

　　大力发展民营经济。实施民营经济腾飞计划，推进股票发行注册制、公司注册资本认缴登记制，放宽市场准入，降低经营成本，加快科技型、成长型、外向型企业发展。实施中小微企业成长工程，实行普惠制创业辅导，健全融资担保机制，完善专项资金引导，形成全民创业热潮。

　　3、壮大县区经济实力。县区生产总值、公共财政预算收入、固定资产投资平均增速超过全市2个百分点以上，努力推动县区经济成为全市经济的主要支撑力量。

　　加快县域经济发展。完善3个县城功能规划，扩大城镇化空间。挖掘乡村文化内涵，打造沙后所、虹螺岘等10个明星城镇。加大农业科技投入，稳定粮食作物种植面积，确保丰产丰收。实施新建和改建设施蔬菜、节水滴灌、粮油高产示范区、现代海洋牧场4个“20万亩工程”，建设兴城花生、绥中贝类2个农产品出口示范区。兴城馨予副食品、建昌绿宝露饮料等5个10亿元以上农产品深加工项目建成投产。创新农业生产经营体制，推进农村土地经营权流转，增强农民专业合作组织带动能力，培育家庭农场，拓宽农民增收渠道。

　　加快城区经济发展。重点在CBD和兴城邴家湾一带，建设“十里银湾”集聚带，打造“金岸龙湾”旗帜型服务业集聚区的核心区。实施亿元以上服务业项目40个，推进龙湾大街、五里河两岸和兴城海滨服务业集聚区建设，投资40亿元的隆东国际城市综合体、投资40亿元的龙港兴隆大家庭和飞天广场人防工程等23个项目开工建设，投资10亿元的银象宁远城、投资8.6亿元的大红门物流商贸城等15个项目竣工营业。加快南票新区开发，促进转型发展。推进房地产业健康发展，房地产开发投资额和商品房销售额实现15%以上的增长。大力发展电子商务和普惠金融，推动现代服务业发展。全市服务业发展实现“两高一点”目标。

　　4、促进文化旅游产业提质升级。整合旅游资源，优化旅游开发，提高旅游效益，积极创建中国优秀旅游目的地城市。全年旅游总收入实现338亿元，增长20%。

　　全面优化文化旅游品牌。完善旅游服务设施，启动国家海洋公园、龙湾海滨旅游码头、葫芦岛游客服务中心等项目。加快景点景区建设，建设徐大堡、老大杖子等4个特色景观旅游名镇名村。启动兴城古城更名宁远城，推进与北京中广集团的合作开发，打造明清特色古城。姜女石遗址公园、华谊兄弟影视基地等6个项目开工建设，首山·丽汤等4个项目竣工营业。加快觉华岛整体开发，培育“海岛人家”旅游基地。提高长城、古城等文化旅游品牌美誉度，争创全国海洋旅游示范县、示范岛和示范景区。

　　全面推进创意产业成长。引进演艺联盟，培育文化企业。规划连山古玩一条街等创意文化街，开工建设北方艺术家村等15个文化创意产业项目。比基尼国际博览中心开工建设，比基尼广场投入使用。举办第4届泳博会、泵博会和首届觉华岛菩提文化博览会，打造文化创意品牌。

　　5、加快美丽葫芦岛建设。加快基础设施建设，完善城市功能。建兴高速国庆节竣工通车，实现全域通高速。改造县级以上公路8条、260公里，提升公路网通行能力。建设新老城区快速干道、龙绣街南延等6条街路，提高主城区通畅水平。推进猴山水库建设，青山水库竣工输水，供水管网改扩建二期和青山水源净配水工程开工建设，启动城市排水管网改造工程，提高城市公共保障水平。

　　优化生态宜居环境。持续开展“我们的城乡·我们的家园”环境综合整治活动，按照环境优美标准，打造5个明星街道、10个美丽城镇示范点。提高环境舒适指数，全面推进国家森林城市创建工作，新增城市绿地面积2130亩，农村造林绿化41万亩，完成12.8万亩干鲜果经济林产业带建设，达到国家森林城市标准。实施青山工程，矿山生态治理1325亩，超坡地还林2.4万亩。实施碧水工程，扩建兴城、建昌和主城区污水处理厂，推进凌河、六股河、烟台河、连山河等流域治理，全面提升水质。实施蓝天工程，推进固定污染源、废气、扬尘油烟等10项治理，推进城区拆小并大供热工程，做好大气污染监测预警，综合治理PM2.5，让葫芦岛海碧天蓝、山清水秀。

　　6、发展社会事业和民生工程。顺应人民群众新期待，回应人民群众新要求，努力增强人民群众幸福感。

　　提升人才、就业和社会保障工作水平。实施人才兴市战略，改革人才引进、选拔、培养和录用机制，营造凝心聚人、人尽其才的浓厚氛围。加强职业技能培训，完成农民科技培训5万人次。完善城乡均等化公共就业服务体系，实名制就业4万人，城镇登记失业率控制在3.8%以内。扩大社保覆盖面，促进养老事业、养老产业发展，养老、医疗、失业、工伤、生育保险参保率稳中有升。

　　发展教育卫生和文化体育事业。促进城乡教育资源合理配置，实现一乡一所标准化中心幼儿园全覆盖，新增1所省级特色高中。整合中职教育资源，渤海船院新校园投入使用。加快县级公立医院改革，完善县乡基层卫生服务体系。建设76家社区书屋，推进社区书屋全覆盖。设立文化产业扶持资金，鼓励民营文化企业发展。推进公共文化服务体系建设，实施广播电视户户通工程。丰富群众体育活动，提高竞技体育水平。

　　今年，将为人民群众办好10件实事：一是改善困难群众住房条件，建设保障性住房800户，新增廉租房租赁补贴200户，完成2235户农村危房改造。二是城乡最低生活保障标准提高10%左右，全面实施城乡居民大病保险。三是发展养老事业，启动500张床位的老年公寓建设，对80至89岁低收入老年人给予每人每月100元高龄津贴。四是为3.8万事业单位中高级专业技术人员提高津补贴标准，超过全省平均水平。五是全面为农村贫困妇女免费进行“两癌”筛查，全面实施农村免费孕前优生检查。六是投资1000万元，对全市280万亩粮食作物病虫害实施统防统治。七是投资1000万元，实施全市粮食作物农业保险全覆盖。八是加快贫困群众脱贫致富步伐，6.8万贫困人口人均收入达到3200元。九是实施“天眼”工程，新建治安视频监控平台20个、治安监控摄像头5000个，增强群众安全感。十是实施农村亮化工程，为全市行政村安装2万盏路灯。

　　7、加强民主法制和精神文明建设。加强民主法制建设。自觉接受市人大的法律监督、工作监督，主动接受市政协的民主监督，虚心接受社会公众和新闻舆论监督，提高人大代表议案、建议、批评、意见和政协委员提案办理质量，诚恳听取和采纳民主党派、工商联、无党派人士和各人民团体的意见建议。坚持用制度管权管事管人，严格依法履行职责，推进政务公开和行政监察，规范行政执法行为。加快法治城市建设，维护社会公平正义。

　　深化文明城市创建活动。开展党的十八届三中全会精神和讲诚信、懂规矩、守纪律宣传活动，加强社会主义核心价值观教育、爱国主义教育、革命传统教育，加强公民思想道德建设，加快创建全国文明城市。深化双拥共建活动，支持部队建设，争创全国双拥模范城五连冠。

　　创新社会管理。健全重大决策社会稳定风险评估机制，强化社区职能建设，提升服务水平，及时就地解决群众合理诉求。创新立体化社会治安防控体系，加强全市公安基础建设，依法防范和惩治违法犯罪。实行最严格的食品药品监管制度，完善监管体制，建立长效机制，确保食品药品安全。深化安全生产管理体制改革，构建隐患排查治理和安全预防控制体系。强化应急管控体系，提高处置公共安全危机和突发事件的能力。

　　8、强化政府自身建设。坚持以党的十八届三中全会精神为指导，深入开展党的群众路线教育实践活动，转变政府职能，提高行政效率。

　　坚持深化改革。按照省政府部署，推进22项改革任务。重点推进机构改革和事业单位分类改革，进一步简政放权；推进财税体制改革，降低债务风险；推进土地集约化经营和户籍制度改革，增强城镇化动力；推进公用事业改革和市场准入制度改革，扩大民营经济发展空间。

　　坚持依法行政。强化法律意识，严格依法履职，规范行政决策，完善行政程序。推进行政执法责任制，规范执法，公正执法，文明执法，实现软环境建设制度化，提高行政服务水平。

　　坚持高效务实。健全深入基层调查研究制度，树立现场办公解决问题的作风，事无巨细，事必躬亲，事不过夜。推行目标责任考核制、过错责任追究制，严格绩效管理，促进责任落实。

　　坚持情系群众。完善直接联系和服务群众制度，深怀爱民之心，恪守为民之责，把群众安危冷暖挂在心上。决策因民而定，发展为民而为，多为基层群众雪中送炭，多为惠民工程锦上添花。

　　坚持廉洁奉公。坚决反对“四风”，严格执行党风廉政建设规定。完善惩治和预防腐败体系建设，严格重大资金和项目的监察监管，坚决落实办公用房、用车等系列制度，公开“三公”经费，树立干部清正、政府清廉、政风清明的良好形象。

　　各位代表！新时代赋予新使命，新征程充满新希望。让我们在以习近平同志为核心的党中央的正确领导下，齐心协力，勇往直前，全面加快沿海经济强市建设，努力创造葫芦岛更加富庶文明幸福的美好明天！
